# Improved Preoperative Diagnosis of Medullary Thyroid Carcinoma Using Dual-Mode Ultrasound Radiomics

**DOI:** 10.3390/cancers18111738

**Published:** 2026-05-26

**Authors:** Luying Gao, Naishi Li, Yu Xia, Liyuan Ma, Yuang An, Jiang Ji, Jionghui Gu, Dingyue Zhang, Nengwen Luo, Yang Cao, Yijian Fan, Yuxin Jiang

**Affiliations:** 1 Department of Ultrasound, State Key Laboratory of Complex Severe and Rare Diseases, Peking Union Medical College Hospital, Chinese Academy of Medical Sciences and Peking Union Medical College, Beijing 100730, China; 2 Department of Endocrinology, Key Laboratory of Endocrinology of National Health Commission, State Key Laboratory of Complex Severe and Rare Diseases, Peking Union Medical College Hospital, Chinese Academy of Medical Sciences and Peking Union Medical College, Beijing 100730, China

**Keywords:** thyroid cancer, radiomics, medually thyroid carcinoma, thyroid nodule, ultrasound

## Abstract

**Background**: Preoperative diagnosis of medullary thyroid carcinoma (MTC) is clinically challenging due to sonographic overlap with other thyroid tumors. To address this, we aimed to develop a multi-vendor, multimodal radiomic framework for accurate MTC identification, comparing its diagnostic performance with that of experienced radiologists. **Methods**: This retrospective study included 467 pathologically confirmed thyroid nodules (94 MTCs, 373 non-MTCs) acquired across multiple ultrasound platforms. The dataset was randomly partitioned into training (80%) and internal testing (20%) sets. In total, 2250 radiomic features were extracted from grayscale and color Doppler images, followed by Z-score normalization to mitigate batch effects. A robust feature selection strategy (LASSO and recursive feature elimination) identified optimal signatures for developing machine learning classifiers (SVM, LR, RF). The optimal model was further validated on an independent, balanced cohort (*n* = 60; comprising 12 cases each of MTC, papillary carcinoma, follicular carcinoma, follicular adenoma, and nodular goiter) and compared with experienced radiologists across seven classification tasks. **Results**: The RF model achieved an AUC of 0.993 in distinguishing MTC from papillary carcinoma. The LR model showed an AUC of 0.991 for identifying MTC from all other nodules. In the independent validation cohort, the models maintained superior discriminatory ability, showing better diagnostic performance compared to the image interpretation by radiologists (AUC 0.993 vs. 0.488, *p* < 0.001). **Conclusions**: The proposed multi-vendor, multimodal radiomic system demonstrated good discriminative ability in the diagnosis and stratification of MTC. By integrating grayscale and Doppler ultrasound features while overcoming scanner variability, this model shows potential as a non-invasive adjunctive tool.

## Simple Summary

Medullary thyroid carcinoma (MTC) is a rare but aggressive type of thyroid cancer. Accurate preoperative diagnosis using standard ultrasound is highly challenging and relies heavily on the doctor’s experience, often leading to misdiagnosis or inadequate surgery. To address this, we developed a non-invasive artificial intelligence (AI) method. Our results demonstrated that this radiomics model showed good discriminative ability in the diagnosis and stratification of MTC. By overcoming the limitations of subjective evaluation and equipment differences, this AI tool shows potential as a non-invasive adjunctive tool.

## 1. Introduction

Thyroid nodules are a prevalent clinical disease. Clinically, they are classified into benign and malignant lesions. The majority of these nodules are benign, primarily comprising nodular goiters (NGs) and follicular adenomas (FAs). Thyroid cancer is the seventh most common malignancy globally, with its incidence steadily rising [1,2]. The vast majority of thyroid cancers are derived from follicular cells, comprising differentiated thyroid carcinomas (DTCs)—specifically papillary thyroid carcinoma (PTC), which accounts for approximately 85–90% of cases, follicular thyroid carcinoma (FTC), accounting for 5–10%, and highly aggressive anaplastic thyroid carcinoma (ATC, <1%) [1]. Medullary thyroid carcinoma (MTC) occupies a unique position within this classification as a neuroendocrine malignancy originating from parafollicular C cells. MTC accounts for only 1–2% of all thyroid cancers [1,2]. Ultrasound serves as the primary modality for thyroid nodule assessment. Malignant nodules are conventionally identified by classic ultrasound features such as solid composition, hypoechogenicity, irregular margins, and microcalcifications [1,2].

Since PTC is typically an indolent tumor with a favorable prognosis, active surveillance has become an accepted management strategy for low-risk patients [3,4]. However, this conservative approach is not appropriate for MTC. MTC exhibits a far more aggressive clinical course [5,6,7]. While ultrasound is effective for PTC, guidelines such as the 2017 American College of Radiology Thyroid Imaging, Reporting and Data System (ACR TI-RADS) struggle to accurately identify MTC [8,9]. Approximately 30% of MTC cases present without classic malignant features, which confounds standard diagnosis. To improve detection, our previous study hypothesized that evaluating the vascular structure of MTC may provide a diagnostic advantage over analyzing morphology alone [10]. In clinical practice, the measurement of serum calcitonin (Ctn) and Ctn in the washout fluid of fine-needle aspiration biopsy (FNA-Ctn) increased the diagnostic accuracy for MTC. However, despite its high sensitivity, routine screening of serum Ctn for thyroid nodules is not universally mandated due to cost-effectiveness debates and varying availability, and FNA is an invasive procedure. Therefore, there is a clinical need for a non-invasive imaging tool to optimize preoperative screening [4].

To overcome these diagnostic limitations, researchers are turning to ultrasound radiomics [11]. Current radiomic applications in thyroid tumors focus predominantly on grayscale images of PTC, while the exploration of MTC remains in its preliminary stages [12]. Moreover, the clinical translation of existing models is often hindered by a reliance on single-vendor datasets, which fail to account for scanner-induced image variations and limit their real-world generalizability.

To address this gap, this study aims to construct a multimodal artificial intelligence classifier that integrates radiomic features from both grayscale and color Doppler ultrasound, thereby enhancing preoperative diagnostic accuracy of ultrasound and clinical adjunctive decision-making. The model was developed utilizing a multi-vendor dataset, and the model’s diagnostic performance was evaluated using an independent, balanced multi-class clinical cohort.

## 2. Materials and Methods

### 2.1. Study Population and Data Acquisition

Patient data were extracted from our center’s ultrasound database from January 2017 to January 2025. We initially reviewed the medical records of all patients who underwent surgical resection and were pathologically diagnosed with medullary thyroid carcinoma (MTC). The inclusion criteria for the MTC group were (a) confirmed diagnosis of MTC via pathology following total or partial thyroidectomy and (b) availability of preoperative thyroid ultrasound examinations performed at our institution. Patients were excluded if (a) preoperative ultrasound data were incomplete, specifically lacking Color Doppler images, or (b) image quality was insufficient for analysis. In total, 106 MTC cases were included.

A control group of non-MTC thyroid tumors was established from the same database through stratified random sampling. The overall data were strategically allocated into two components: a primary development cohort and an independent validation cohort. The primary development cohort consisted of 467 thyroid tumors (94 MTC cases and 373 non-MTC cases, including 98 follicular thyroid carcinomas [FTCs], 99 follicular adenomas [FAs], 88 papillary thyroid carcinomas [PTCs], and 86 nodular goiters [NGs]). This cohort was stratified by pathological type and randomly partitioned into a training set (80%, *n* = 374) for model development and an internal testing set (20%, *n* = 93) for preliminary evaluation. This 4:1 allocation ratio was predetermined based on machine learning heuristics. Given the moderate size of our primary cohort, this split balances the need for a sufficiently large training set while retaining an adequately sized internal test set for reliable preliminary evaluation.

Prior to model evaluation, an independent validation cohort (*n* = 60) was constructed from independent cases not included in the primary cohort. As for morphological differentiation, this cohort was designed with an artificial 1:1 class balance, comprising exactly 12 cases each of MTC, PTC, FTC, FA, and NG. On this independent set, the diagnostic performance of the final model was compared with two experienced radiologists (with 10 and 16 years of experience in thyroid ultrasound, respectively). During the initial evaluation phase, both radiologists independently reviewed the images while remaining blinded to all clinical data, pathology, and each other’s assessments. In cases of disagreement between the two radiologists, a consensus diagnosis was reached through discussion. As they became aware of each other’s assessments during this process, the blinding between observers was conditional.

Ultrasound examinations were performed using the Philips EPIQ Elite or iU22 (Philips Healthcare, Bothell, WA, USA), GE Logiq E9/E10 (GE Healthcare, Milwaukee, WI, USA), and Mindray Resona 7 (Mindray Bio-Medical Electronics, Shenzhen, China). All systems were equipped with high-frequency linear-array transducers (ranging from 5 to 15 MHz). Standardized scanning protocols were followed to optimize imaging parameters for each patient. Representative grayscale and Color Doppler images were exported for subsequent radiomic analysis.

### 2.2. Image Segmentation and Feature Extraction

Regions of interest (ROIs) were initially generated via automatic segmentation on the ultrasound slice exhibiting the maximum nodule diameter. To ensure strict anatomical precision, all boundaries were manually verified and refined by two experienced radiologists (>5 years of experience in thyroid imaging).

Standardized image preprocessing was performed to mitigate inter-scanner variability. Images were resampled to an isotropic pixel spacing of 1.0 × 1.0 mm using B-spline interpolation, and grayscale intensities were discretized using a fixed bin width of 25.

Radiomic feature extraction was executed using the PyRadiomics package (version 3.0.1). A total of 2250 quantitative features were extracted per ROI, encompassing first-order statistics, shape-based metrics, and advanced texture features derived from Gray Level Co-occurrence (GLCM), Run Length (GLRLM), Size Zone (GLSZM), and Dependence Matrices (GLDM). Wavelet filters were applied to capture multi-scale spatial information.

To standardize the data scale and explicitly mitigate vendor-specific batch effects across different ultrasound platforms, all extracted features underwent robust Z-score normalization prior to the feature selection phase.

### 2.3. Feature Selection and Model Development

To manage the high-dimensional feature space and mitigate the risk of overfitting, a rigorous two-stage feature selection strategy was implemented exclusively on the training set. In the preliminary stage, L1-regularization (LASSO) was applied to linear classifiers (Logistic Regression and Support Vector Machine), retaining features with non-zero coefficients based on an optimal penalty parameter determined via logarithmic grid search (10^0^ to 10^6^) and cross-validation. Conversely, for the Random Forest classifier, features were pre-filtered based on mean importance scores.

In the second stage, Recursive Feature Elimination with Cross-Validation (RFECV) was employed on the retained subsets to identify the optimal feature combination that maximized diagnostic accuracy, while isolating the most robust and highly discriminative radiomic signatures. The final radiomic signature was restricted to a maximum of 14 features. Three classifiers—Support Vector Machine (SVM), Logistic Regression (LR), and Random Forest (RF)—were developed using the training set. We performed an inter-observer reproducibility analysis to validate the robustness of the features utilized in our final model. A subset of 30 randomly selected cases was independently segmented by two radiologists blinded to each other. The Intraclass Correlation Coefficient (ICC) was calculated and robust features (ICC > 0.75) were retained for final modeling.

**Figure 1:**
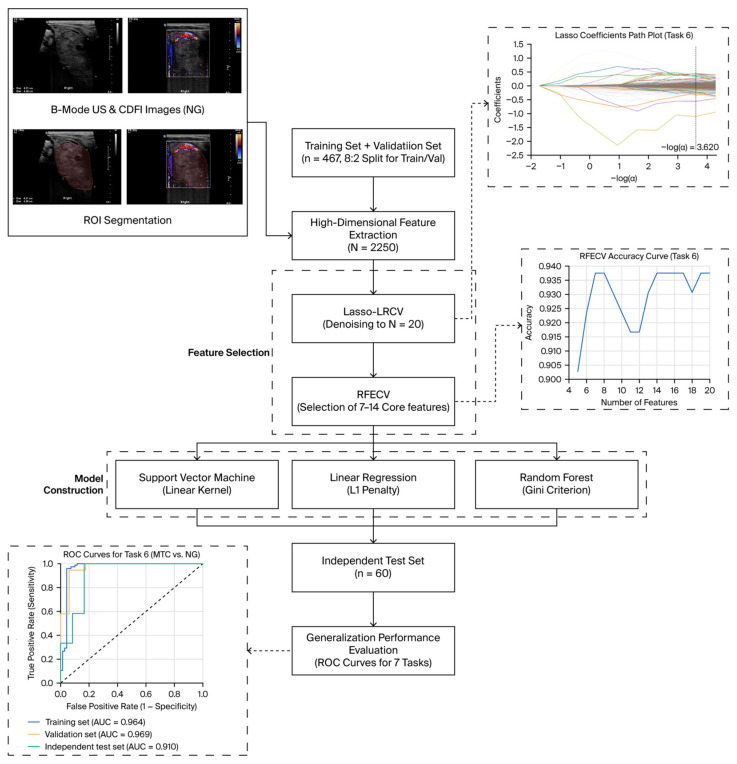
Flow diagram of the radiomics workflow. Representative B-mode ultrasound and color Doppler flow imaging (CDFI) images of nodular goiter (NG) are shown in the upper-left panel. The red contour/shaded area indicates the automatically segmented region of interest (ROI) of the thyroid nodule. The upper-right panel shows the LASSO coefficient path plot for Task 6, in which each colored curve represents the coefficient trajectory of an individual radiomic feature, and the vertical dashed line indicates the selected regularization parameter. The lower-right panel shows the RFECV accuracy curve, where the blue line represents the cross-validated accuracy according to the number of selected features. The lower-left panel shows the ROC curves for Task 6, with blue, orange, and green curves representing the training set, validation set, and independent test set, respectively; the black dashed diagonal line represents the reference line for random classification. Abbreviation: RFECV: Recursive Feature Elimination with Cross-Validation; NG: Nodular Goiter; ROC, Receiver operating characteristic; ROI, Region of interest.

To prevent data leakage, all feature selection and hyperparameter tuning were conducted exclusively within the training set. This strict pipeline ensured that the independent validation cohort remained strictly isolated for final performance evaluation (Figure 1).

### 2.4. Statistical Analysis

Statistical analysis was performed using Python version 3.11 (Python Software Foundation, Wilmington, DE, USA). The diagnostic performance of the models was evaluated using receiver operating characteristic (ROC) curve analysis. Key metrics, including the area under the curve (AUC), sensitivity, specificity, and accuracy, were calculated with their 95% confidence intervals (CIs). To assess the agreement between predicted probabilities and observed outcomes, model calibration was evaluated using calibration curves and the Hosmer–Lemeshow goodness-of-fit test. Additionally, clustered heatmaps were generated to visualize the discriminative patterns of the selected radiomic features. The DeLong test was utilized to statistically compare the differences in AUCs between the models and the radiologists. A two-sided *p*-value < 0.05 was considered statistically significant. All model development and evaluation procedures were implemented using the scikit-learn library.

## 3. Result

### 3.1. Radiomic Feature Identification and Signature Verification

**Figure 2:**
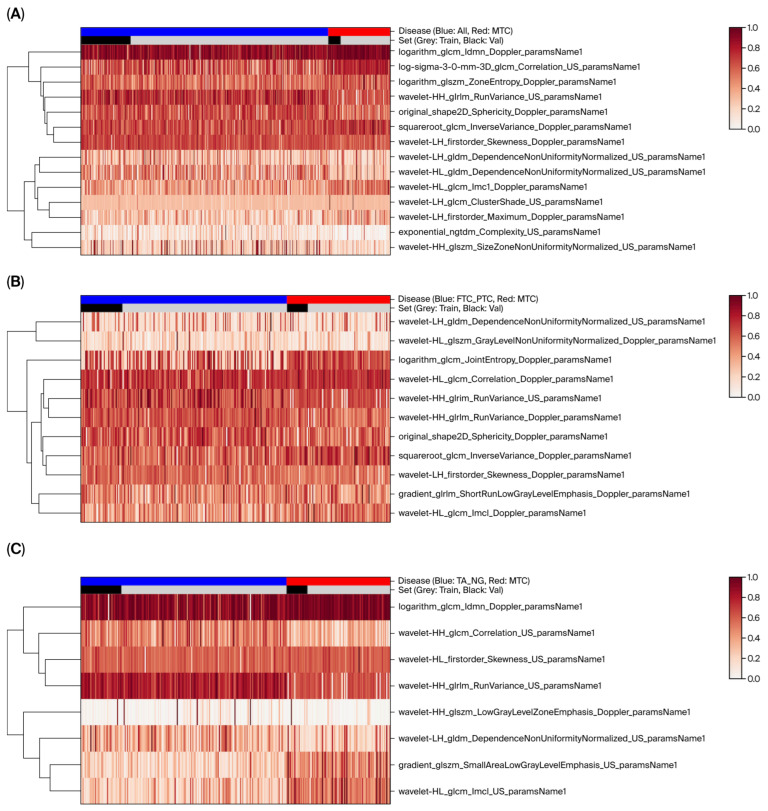
Unsupervised hierarchical clustering heatmaps of selected radiomic features (**A**) MTC vs. All Non-MTC nodules, (**B**) MTC vs. Other Malignancies, (**C**) MTC vs. Benign Thyroid Nodules. Abbreviation: MTC: Medullary Thyroid Carcinoma.

The sequential application of L1-regularization (or importance-based filtering) and RFECV reduced the initial dimensionality of 2250 variables to robust, task-specific feature subsets. These selected features demonstrated strong independent discriminative capability, as evidenced by the unsupervised hierarchical clustering analysis shown in Figure 2. The heatmaps reveal clear phenotypic segregation, with MTC cases (red annotation) and control groups (blue annotation) forming distinct clusters based solely on their radiomic expression profiles. This separation confirms that the identified signatures capture the intrinsic morphological heterogeneity of MTC, providing a solid biological basis for the subsequent machine learning classification.

### 3.2. Differentiation of MTC from Other Malignant Thyroid Tumors

**Figure 3:**
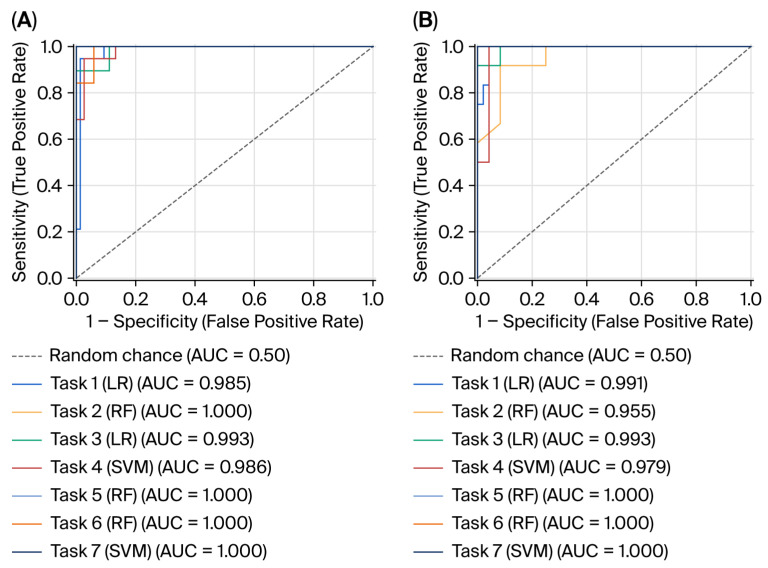
(**A**) ROC Curves for the Internal Validation Set. (**B**) ROC Curves for the Independent Validation Set. Task 1: MTC vs. Non-MTC (FTC + PTC + NG + FA); Task 2: MTC vs. FTC; Task 3: MTC vs. PTC. Task 4: MTC vs. other malignancies (FTC + PTC); Task 5: MTC vs. FA; Task 6: MTC vs. NG; Task 7: MTC vs. Benign tumors (NG + FA). Abbreviation: MTC: Medullary Thyroid Carcinoma; FA: Follicular Adenoma; NG: Nodular Goiter; FTC: Follicular Thyroid Carcinoma; PTC: Papillary Thyroid Carcinoma; RF: Random Forest; SVM: Support Vector Machine; ROC: Receiver Operating Characteristic; LR: Logistic regression.

In the task of differentiating MTC from other malignant thyroid tumors, all three machine learning classifiers—SVM, LR, and RF—demonstrated strong discriminative performance in distinguishing MTC from FTC and PTC. The performance of the models is summarized in Table 1 and Figure 3.

For the combined task of distinguishing MTC from a pooled group of other malignancies (FTC and PTC combined), the SVM was identified as the optimal classifier. The SVM model achieved a validation AUC of 0.986 (95% CI: 0.964–1.000), with an accuracy, sensitivity, and specificity of 0.947.

For the differentiation between MTC and FTC, the RF model yielded the highest diagnostic efficacy. In the validation cohort, the RF model achieved discrimination with an AUC of 1.000 (95% CI: 1.000–1.000), with an accuracy, sensitivity, and specificity of 1.000.

In the differentiation between MTC and PTC, the LR model proved to be the most effective classifier. It achieved a validation AUC of 0.988 (95% CI: 0.966–1.000), with an accuracy of 0.946, sensitivity of 0.895, and specificity of 1.000.

### 3.3. Differentiation of MTC from Benign Thyroid Tumors

Following the assessment, we evaluated the performance of the machine learning classifiers in distinguishing MTC from benign thyroid pathologies (FA and NG). The performance of the models is detailed in Table 1 and Figure 3.

For the task of distinguishing MTC from combined benign nodules (FA and NG), the SVM was identified as the most robust classifier. The SVM model achieved good diagnostic performance in the validation set, with an AUC of 1.000 (95% CI: 1.000–1.000) and an accuracy, sensitivity, and specificity of 1.000.

For the specific differentiation between MTC and FA, the RF model achieved optimal performance. It demonstrated good classification capabilities in the validation cohort, yielding an AUC of 1.000 (95% CI: 1.000–1.000) with 100% accuracy, sensitivity, and specificity.

In differentiating MTC from NG, the RF model again outperformed other classifiers. It attained a validation AUC of 0.991 (95% CI: 0.970–1.000), with an accuracy of 0.972, sensitivity of 1.000, and specificity of 0.941.

### 3.4. Comprehensive Differentiation of MTC from Other Thyroid Nodules

We further assessed the models’ ability to distinguish MTC from a pooled cohort of all other thyroid nodules, including both malignant (FTC, PTC) and benign (FA, NG) tumors.

Among the evaluated algorithms, the LR classifier emerged as the optimal model for this comprehensive differentiation task. As detailed in Table 1 and Figure 3, the LR model achieved an AUC of 0.985 (95% CI: 0.963–1.000), with an accuracy of 0.979, a sensitivity of 0.947, and a specificity of 0.987.

### 3.5. Performance Comparison with Radiologists on the Independent Validation Set

To validate clinical generalizability, the optimal models were tested on an independent cohort of 60 cases and compared with experienced radiologists. The models showed a higher diagnostic accuracy compared to the image interpretation by experienced radiologists across all seven classification tasks (*p* < 0.05). The performance of the models is detailed in Table 2 and Figure 3. In the comprehensive assessment distinguishing MTC from all other thyroid nodules, the LR model achieved an AUC of 0.991, compared to the radiologists’ performance of 0.579.

Similarly, for the combined task against other malignancies, the SVM model showed a higher diagnostic accuracy compared to the image interpretation by experienced radiologists (AUC 0.979 vs. 0.546). For MTC vs. FTC, the RF model achieved an AUC of 0.955 compared to 0.729 for radiologists. For MTC vs. PTC, the RF model achieved an AUC of 0.993 compared to 0.488 for radiologists. In differentiating MTC from benign pathologies, the SVM model yielded an AUC of 1.000, compared to the radiologists’ AUC of 0.687. The RF model attained an AUC of 1.000 for distinguishing MTC from FA or NG, while radiologists achieved an AUC of 0.729 for both tasks.

### 3.6. Robustness and Calibration of Predictive Models

**Figure 4:**
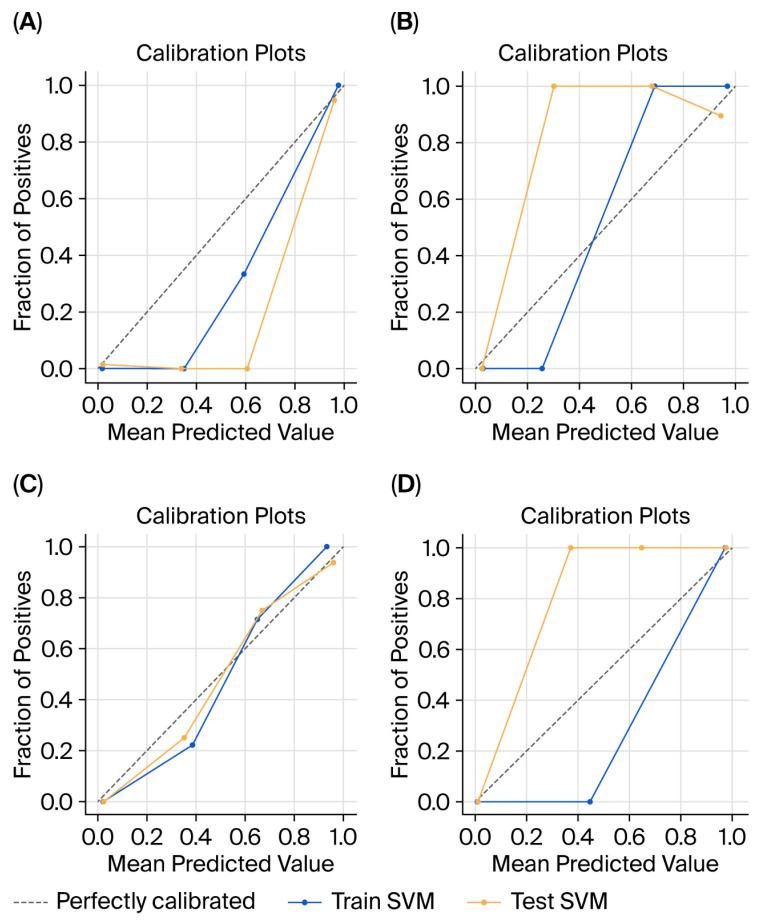
The calibration curve for the best-performing model. (**A**) MTC vs. Non-MTC (**B**) MTC vs. PTC (**C**) MTC vs. Other Malignancies (**D**) MTC vs. Benign Thyroid Nodules. Abbreviation: MTC: Medullary Thyroid Carcinoma; PTC: Papillary Thyroid Carcinoma; SVM: Support Vector Machine; LR: Logistic regression.

To rigorously evaluate the generalizability and clinical reliability of the proposed radiomic signatures, an assessment of overfitting and calibration performance was conducted. The models demonstrated exceptional robustness, maintaining AUC values between 0.910 and 1.000 in the independent validation test cohorts across all seven classification tasks, with minimal performance degradation compared to the training set. This stability confirms the absence of severe overfitting and suggests strong potential for clinical application. Calibration analysis further validated the accuracy of risk probability estimation. The best-performing models for each task consistently passed the Hosmer–Lemeshow goodness-of-fit test (*p* > 0.05), with calibration curves demonstrating high concordance between predicted malignancy risks and observed pathological outcomes (Figure 4).

### 3.7. Multimodal Feature Interpretation and Contribution Analysis

**Figure 5:**
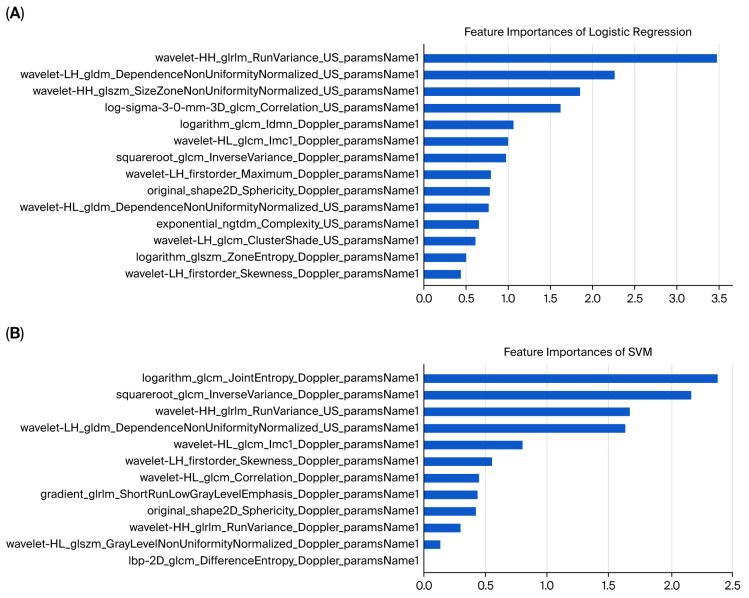
Feature importance analysis illustrating the complementary value of multimodal radiomics. (**A**) MTC vs. Non-MTC (**B**) MTC vs. Other Malignancies. Abbreviation: SVM: Support Vector Machine.

To elucidate the biological basis of the model’s decision-making process, we analyzed the feature importance weights across different classification tasks. For the general task (MTC vs. Non-MTC), grayscale ultrasound features played a dominant role. Specifically, texture features such as wavelet-HH_glrlm_RunVariance_US exhibited the highest contribution weights (Figure 5A). For the subtype differentiation task (MTC vs. Other Malignancies), the contribution of color Doppler flow features increased significantly. As shown in Figure 5B, Doppler-derived features, notably logarithm_glcm_JointEntropy_Doppler, emerged as the top predictors.

## 4. Discussion

The preoperative diagnosis of MTC presents a diagnostic dilemma due to its rarity and sonographic overlap with both benign nodules and other malignancies. In this study, we developed a multimodal radiomic framework, which achieved high diagnostic precision. In the independent validation cohort, the radiomics models demonstrated a higher diagnostic capacity compared to the image interpretation by radiologists. This difference was most pronounced in the task of differentiating MTC from PTC. While the radiologists’ image evaluation yielded an AUC of 0.488, the Random Forest model maintained discriminative ability (AUC 0.993). Consistent with this trend, in distinguishing MTC from all other thyroid nodules, the Logistic Regression model (AUC 0.991) also yielded higher accuracy than the radiologists’ image assessment (AUC 0.579). These results suggest that quantitative radiomics may identify patterns specific to MTC. However, it is crucial to interpret this comparison with caution. The baseline diagnostic performance of the radiologists in this study was evaluated solely on static ultrasound images, strictly blinded to essential clinical and biochemical data, most notably serum calcitonin levels.

While deep learning and radiomics have been applied to thyroid nodule diagnosis, the majority of studies focus on the binary distinction between benign nodules and PTC [13,14]. Research specifically targeting MTC remains scarce. Zhang et al. reported a radiomic AUC of 0.93 for differentiating MTC from PTC [12]. The performance leap of our model (AUC > 0.95) is likely attributable to our comprehensive inclusion of multimodal data. Unlike prior works restricted largely to B-mode imaging, we incorporated Color Doppler flow features, which help distinguish MTC from other carcinomas.

The dominance of grayscale ultrasound features in distinguishing MTC from benign nodules aligns with the pathological characteristics of thyroid cancer. Biologically, MTC is characterized by the presence of amyloid deposition, stromal fibrosis, and coarse calcifications [15]. Notably, the feature with the highest contribution weight was wavelet-HH_glrim_RunVariance_US. In texture analysis, RunVariance measures the variance in runs of pixels with the same grey level, serving as an indicator of local heterogeneity. Biologically, this reflects the microenvironment of MTC. These microscopic heterogeneities create complex acoustic scattering patterns that are subtle to the human eye but are effectively captured by radiomic extraction. As firmly supported by our earlier data, specifically the contribution weights of B-mode features in the model and the resulting high accuracy (AUC > 0.99) in the independent test set, this fundamental structural disruption is best reflected in the grayscale textural profile.

In distinguishing MTC from other malignancies (PTC/FTC), the contribution of color Doppler flow features increased significantly. MTC, being a neuroendocrine tumor, typically exhibits a rich, chaotic vascular network driven by specific angiogenic factors [15,16]. This contrasts with PTCs, which often manifest as hypovascular or exhibit specific peripheral flow patterns [17]. The high predictive value of Doppler features captured this vascular chaos. This finding suggests that while grayscale confirms cancer, Doppler defines the type of cancer, highlighting the necessity of multimodal data integration for precise diagnosis.

Current guidelines, such as the ACR TI-RADS and ATA guidelines, are primarily optimized for PTC detection and often result in indeterminate classifications for MTC due to its atypical presentation [8,18,19]. This diagnostic uncertainty often leads to inadequate surgical planning. The high negative predictive value of our model suggests that it may serve as an effective “rule-out” tool to reduce unnecessary biopsies, while its high specificity provides the confidence for aggressive surgical interventions.

As observed in our calibration curves (Figure 4), the models exhibited varying degrees of calibration accuracy. Model C (Logistic Regression) showed a reasonable approximation to the ideal 45° line. In contrast, models corresponding to panels A, B, and D showed deviation. This is a phenomenon where algorithms optimized for margin-based classification or hierarchical splitting focus on maximizing discriminative boundaries rather than estimating true clinical probabilities, leading to risk overestimation or underestimation at the extremes.

The reliability of our models relied on hyperparameter optimization and techniques like RFECV to prevent overfitting, a vital principle similarly demonstrated in recent physiological AI modeling [20]. Furthermore, while our framework utilized hand-crafted radiomics, future research may explore other deep learning architectures (e.g., CNNs, Vision Transformers, and YOLO), which have shown automated feature extraction capabilities in other medical imaging domains [21].

There were several limitations to our study. First, given the retrospective design at a single regional referral center, inherent selection bias exists. Second, ROI segmentation, though semi-automated, still relied on manual correction. While future iterations aim for fully automated segmentation, current algorithms struggle with the poorly defined margins typical of MTC, meaning semi-automated approaches will likely remain the reliable standard in the near term. Third, although Z-score normalization and standardized protocols were applied, we did not utilize batch-effect correction techniques like ComBat harmonization, which may be addressed in future. Fourth, the reported high AUC of 0.99 should be interpreted prudently. Driven by the relatively limited sample size and an artificial 1:1 class balance designed for morphological proof-of-concept, these metrics likely represent inflated performance estimates. Consequently, the model remains susceptible to overfitting, and its PPV may decrease when applied to a real-world screening population with a lower disease prevalence. Thus, this model may be viewed as an adjunctive decision-support system targeted at suspicious nodules. Independent, multi-center external validation on unselected cohorts is essential to confirm the algorithm’s true generalizability.

## 5. Conclusions

In conclusion, we present a comprehensive radiomic model for the diagnosis and stratification of MTC. By integrating the patterns of grayscale and color Doppler ultrasound, the proposed model shows potential as a non-invasive adjunctive tool. Moreover, this approach offers an interpretable biological basis for AI-assisted MTC management.

## Figures and Tables

**Table 1:** Differentiation of Medullary Thyroid Carcinoma from Thyroid Tumors in the Internal Validation Set.

Classification Task	Model	AUC of Training Set	AUC (95% CI) of Validation Set	Accuracy of Validation Set	Sensitivity of Validation Set	Specificity of Validation Set
MTC vs. Non-MTC (FTC + PTC + NG + FA)	SVM	1.000	0.985(0.962, 1.000)	0.979	0.947	0.987
	LR	1.000	0.985(0.963, 1.000)	0.979	0.947	0.987
	RF	1.000	0.987(0.969, 1.000)	0.957	0.947	0.960
MTC vs. FTC	SVM	1.000	0.992(0.974, 1.000)	0.974	1.000	0.950
	LR	1.000	0.992(0.974, 1.000)	0.949	1.000	0.900
	RF	1.000	1.000(1.000, 1.000)	1.000	1.000	1.000
MTC vs. PTC	SVM	1.000	0.980(0.946, 1.000)	0.946	1.000	0.889
	LR	1.000	0.988(0.966, 1.000)	0.946	0.895	1.000
	RF	1.000	0.988(0.966, 1.000)	0.946	0.895	1.000
MTC vs. other malignancies (FTC + PTC)	SVM	0.999	0.986(0.964, 1.000)	0.947	0.947	0.947
	LR	0.999	0.988(0.968, 1.000)	0.930	1.000	0.895
	RF	1.000	0.969(0.932, 1.000)	0.912	0.895	0.921
MTC vs. FA	SVM	1.000	1.000(1.000, 1.000)	1.000	1.000	1.000
	LR	1.000	1.000(1.000, 1.000)	0.974	1.000	0.950
	RF	1.000	1.000(1.000, 1.000)	1.000	1.000	1.000
MTC vs. NG	SVM	0.968	0.978(0.939, 1.000)	0.944	0.947	0.941
	LR	0.964	0.969(0.916, 1.000)	0.944	0.947	0.941
	RF	1.000	0.991(0.970, 1.000)	0.972	1.000	0.941
MTC vs. Benign tumors (NG + FA)	SVM	1.000	1.000(1.000, 1.000)	1.000	1.000	1.000
	LR	1.000	1.000(1.000, 1.000)	1.000	1.000	1.000
	RF	1.000	1.000(1.000, 1.000)	1.000	1.000	1.000

Abbreviation: MTC: Medullary Thyroid Carcinoma; FA: Follicular Adenoma; NG: Nodular Goiter; FTC: Follicular Thyroid Carcinoma; PTC: Papillary Thyroid Carcinoma; RF: Random Forest; SVM: Support Vector Machine; AUC: Area Under the Receiver Operating Characteristic Curve; CI: Confidence Interval; LR: Logistic regression.

**Table 2:** Model Performance Comparison with Radiologists on the Independent Validation Set.

Classification Task	Model	AUC (95% CI)	Accuracy	Sensitivity	Specificity
MTC vs. Non-MTC (FTC + PTC + NG + FA)	LR	0.991 (0.976, 1.000)	0.950	1.000	0.938
	Radiologists	0.579 (0.360, 0.744)	0.783	0.167	0.938
MTC vs. FTC	RF	0.955 (0.880, 1.000)	0.917	0.917	0.917
	Radiologists	0.729 (0.307,0.777)	0.542	0.167	0.917
MTC vs. PTC	LR	0.993 (0.974, 1.000)	0.917	0.917	0.917
	Radiologists	0.488 (0.351,0.816)	0.583	0.167	1.000
MTC vs. other malignancies (FTC + PTC)	SVM	0.979 (0.937, 1.000)	0.972	1.000	0.958
	Radiologists	0.546 (0.355, 0.770)	0.694	0.167	0.958
MTC vs. FA	RF	1.000 (1.000, 1.000)	1.000	1.000	1.000
	Radiologists	0.729 (0.307,0.777)	0.542	0.167	0.917
MTC vs. NG	RF	1.000 (1.000, 1.000)	1.000	1.000	1.000
	Radiologists	0.729 (0.307,0.777)	0.542	0.167	0.917
MTC vs. Benign tumors (NG + FA)	SVM	1.000 (1.000, 1.000)	1.000	1.000	1.000
	Radiologists	0.687 (0.335, 0.748)	0.667	0.167	0.917

Abbreviation: MTC: Medullary Thyroid Carcinoma; FA: Follicular Adenoma; NG: Nodular Goiter; FTC: Follicular Thyroid Carcinoma; PTC: Papillary Thyroid Carcinoma; RF: Random Forest; SVM: Support Vector Machine; AUC: Area Under the Receiver Operating Characteristic Curve; CI: Confidence Interval; LR: Logistic regression.

## Data Availability

The datasets generated and/or analyzed are available from the corresponding authors on reasonable request.
